# Prevalence and Correlates of Prenatal Depression, Anxiety and Suicidal Behaviours in the Volta Region of Ghana

**DOI:** 10.3390/ijerph18115857

**Published:** 2021-05-29

**Authors:** Nuworza Kugbey, Martin Ayanore, Phidelia Doegah, Masauso Chirwa, Susan A. Bartels, Colleen M. Davison, Eva Purkey

**Affiliations:** 1 Department of General Studies, School of Natural and Environmental Sciences, University of Environment and Sustainable Development, Somanya PMB, Ghana; 2 Department of Health Policy Planning and Management, School of Public Health, University of Health and Allied Sciences, Ho PMB 31, Ghana; mayanore@uhas.edu.gh; 3 Institute of Health Research, University of Health and Allied Sciences, Ho PMB 31, Ghana; tdoegah@uhas.edu.gh; 4 Department of Social Work and Sociology, University of Zambia, Lusaka 10101, Zambia; chirwamas@gmail.com; 5 Department of Public Health Sciences, Queen’s University, Kingston, ON K7L 3N6, Canada; susanabartels@gmail.com (S.A.B.); davisonc@queensu.ca (C.M.D.); 6 Department of Emergency Medicine, Queen’s University, Kingston, ON K7L 4V7, Canada; 7 Department of Global Development Studies, Queen’s University, Kingston, ON K7L 3N6, Canada; 8 Department of Family Medicine, Queen’s University, Kingston, ON K7L 3G2, Canada; eva.purkey@queensu.ca

**Keywords:** pregnancy, depression, anxiety, suicidal behaviors, Ghana

## Abstract

Pregnancy is associated with several physical and psychosocial challenges that influence women’s health and wellbeing. However, prenatal mental health has received little attention. Therefore, this study examined the prevalence and correlates of prenatal depression, anxiety and current suicidal behaviors among pregnant women in the Volta Region of Ghana. Two hundred and fourteen (*n* = 214) pregnant women recruited from two hospitals responded to the hospital depression and anxiety scale (HADS), the insomnia severity index, and a set of psycho-behavioral, socioenvironmental and demographic characteristic questions. Chi-squared, bivariate and multivariate logistic regression were used for data analysis. Prevalence of prenatal depression, anxiety and current suicidal behaviors was 50.5%, 35.5% and 3.3%, respectively. After controlling for other variables, average monthly income, insomnia, non-nutritious food consumption (pica), and body image satisfaction were significantly associated with depression. Marital status, insomnia, lifetime suicidal behavior and partner support were significantly associated with anxiety. Current partner abuse was the only factor significantly associated with current suicidal behavior. The high prevalence rates of anxiety and depression among pregnant women and intimate partner violence remain important maternal health issues in the region. Therefore, brief mental health screening and counseling services should be integrated into prenatal healthcare services.

## 1. Introduction

Mental health problems during pregnancy have significant impacts on women’s health, both during pregnancy and in the postpartum period. Mother’s prenatal mental health has been associated with child outcomes, such as poor birth outcomes, including poor fetal growth, low birth weight, infant diarrheal morbidity, breastfeeding problems, maternal-child interactions, and lower cognitive development [1,2,3]. Prenatal affective disorders, such as depression and anxiety, are the most commonly reported mental health problems among pregnant women [3,4].

Affective disorders are some of the most researched and commonly reported problems among pregnant women. Evidence suggests that prenatal depression and anxiety rates are lower in high-income countries and relatively higher among pregnant women in low and middle-income countries [3,4,5,6]. For example, studies conducted in some Sub-Saharan African (SSA) countries found similar patterns of prenatal depression rates; Malawi-19% [7], Ethiopia-23% and 26% pooled prevalence [8] and Nigeria-24% [9]. However, there are some discrepancies in the prevalence rates in low-income and high-income countries (LMICs) as a systematic review, including studies from thirty high-, middle- and low-income countries, found the prevalence of prenatal anxiety and depression to be less than 10% with no country-specific differences [10]. In a study conducted among a sample of rural Ghanaian pregnant women (21,135), a relatively low prevalence rate of 9.9% prenatal depression was found [3]. Overall, studies in LMICs indicate that between 1 and 3 in 10 women experience affective disorders during pregnancy.

Suicidal behaviors contribute to maternal morbidity and mortality. However, there is little attention in the empirical literature on the burden of suicidal behaviors among pregnant women. Studies have reported varied rates of suicidal behavior, including ideation/thoughts, plans and attempts. For example, in a study among 376 pregnant women in South Africa, 18% of the participants reported suicidal ideations and behaviors [11]. In a recent study among 2062 pregnant women in Peru, 22.6% reported current suicidal behaviors, 22.4% reported a lifetime history of suicidal ideation, 7.2% reported a history of planning suicide, and 6.0% had attempted suicide [12]. These high prevalence rates of suicidal behaviors were significantly predicted by the presence of comorbid mental health problems, such as depression, anxiety and sleep problems [11,13], history of childhood abuse [12,14,15], lack of social support, intimate partner violence and unplanned pregnancies [16].

Although suicidal behaviors among pregnant women are rising [12,15,17] and contribute to negative pregnancy outcomes, including maternal mortality [11,18], there is sparse empirical evidence on suicidal behaviors during pregnancy. The prevalence of suicidal behaviors among pregnant women in low-income countries has been reported to be as high as 33% [11,17], and these behaviors pose a significant public health threat.

Some psychosocial and interpersonal risk and protective factors for prenatal mental health problems have been identified in the extant literature. For example, lack of social support (including partner support), unplanned pregnancy, pregnancy complications, pregnancy loss, presence of other mental illnesses, lack of economic power, history of abuse or of domestic violence, and stress are significant risk factors for prenatal anxiety and depression [4,7,8,9,19]. In addition to these psychosocial and interpersonal risk factors, sociodemographic characteristics, such as maternal age, marital status, nulliparous status, education, family size, and income, are also identified as significant risk factors for prenatal anxiety and depression as well as psychological distress [9,19,20,21].

Studies have also reported dissatisfied body image [22], insomnia [23] and substance use as potential risk factors for prenatal mental health problems, such as depression and anxiety. For example, body image dissatisfaction was significantly associated with prenatal depression with pregnant women, who reported less satisfaction with their bodies experiencing more depressive symptoms [24]. In a systematic review, strong evidence of an association between poor sleep and perinatal depression was reported [25]. In a study among pregnant girls in Kenya, it was observed that substance use significantly was significantly associated with depression [26]. These findings have demonstrated the impact of psycho-behavioral factors on prenatal mental health problems.

To address prenatal mental health problems and prevent subsequent adverse health outcomes for pregnant women and their babies, studies focusing on risk and protective factors for depression, anxiety and suicidal behaviors are warranted to inform evidence-based interventions. Depression, anxiety and suicidal behaviors were chosen as they are the commonly reported mental health problems during the prenatal period. Given the global health risks and consequences of prenatal mental health problems, context-specific studies are needed in Ghana to provide empirical evidence for the best strategies to improve prenatal health outcomes. The Volta region was chosen because it is our catchment area and resource limitations on the part of researchers. It is hoped that this study will be replicated across the country to provide a wider perspective on Ghana’s maternal mental health burden.

Informed by the socio-ecological model [27], this study examined the prevalence of prenatal depression, anxiety and suicidal behaviors (ideation, plan and attempt) and their correlates to address this literature gap. The correlates include factors from multifaceted levels that influence pregnant women’s health and wellbeing based on previous literature.

## 2. Materials and Methods

### 2.1. Research Design and Participants

A cross-sectional study design was employed. Women attending antenatal clinics (ANCs) at the Ho Teaching Hospital and the Hohoe Municipal Hospital were conveniently sampled. The two hospitals provide maternal healthcare for women with diverse socioeconomic and cultural backgrounds across the region. The Ho Teaching Hospital is the largest in the region and serves as the regional and one of the teaching hospitals in Ghana located in Ho. The Hohoe Hospital is one of the municipal Hospitals in the Volta region. A total of 214 women were recruited using an acceptable formula [28] for the required minimum sample size at 5% absolute precision and 95% Confidence Interval.

(1) $$n=\frac{{\left(Z_{\frac{\alpha }{2}}\right)}^{2}\times p\left(1-p\right)}{d^{2}}$$

where *n* = required sample size.

$${\left(Z_{\alpha /2}\right)}^{2}=\mathrm{reliability}\;\mathrm{co}\text{-}\mathrm{efficient},\;p=\mathrm{proportion}\;\mathrm{of}\;\mathrm{study}\;\mathrm{population}$$

where *n* = required sample size;

$${\left(Z_{\alpha /2}\right)}^{2}=\;\mathrm{reliability}\;\mathrm{co}\text{-}\mathrm{efficient},\;p=\mathrm{proportion}\;\mathrm{of}\;\mathrm{study}\;\mathrm{population};$$

$$d^{2}=\mathrm{margin}\;\mathrm{of}\;\mathrm{error};$$

$Z_{\alpha /2}=1.96\;\mathrm{at}\;95\%\;\mathrm{confidence}\;\mathrm{level}$;

p=9.9% or 0.099 prevalence of prenatal depression reported by Weobong et al. (2014) in Ghana;

$d^{2}=5\%\;\mathrm{or}\;0.05$.

(2) $$n=\frac{1.96^{2}\times 0.099\left(1-0.0999\right)}{0.05^{2}}=137$$

n=137

Thus, the minimum sample size required was 137 pregnant women. See Table 1 for detailed descriptions of the study participants.

### 2.2. Measures

*Depression and Anxiety* were measured using the hospital anxiety and depression scale [29]. This scale consists of 14 items, which measure depression and anxiety in hospital settings. Each domain has 7 items measured on a 4-point Likert scale scored from 0 (not at all) to 3 (very often indeed). The total score on each subscale ranged between 0 and 21 for anxiety and depression. A cutoff score of ≥8/21 was used to classify “caseness” for depression and anxiety, respectively (Bjelland et al. 2002). In this study, the scale had moderate reliability with Cronbach’s alpha of 0.77 (total scale—HADS), 0.73 for the anxiety subscale and 0.63 for the depression subscale, respectively.

Suicidal behaviors (ideation, plan and attempt) were measured with single items examining both lifetime and current suicidal behaviors developed by the researchers based on a review of prior literature. Items included: “Have you ever had any thoughts of killing yourself in your lifetime?”, “Have you ever made plans of killing yourself in your lifetime?” and “Have you ever attempted killing yourself in your lifetime?”. A two-point response format (“yes”—1 and “no”—0) was used for lifetime questions. A composite score was obtained by adding the three items (0–3). A score of 1 or more represented the presence of a lifetime suicidal behavior. Current suicidal behavior questions included: “In the past one month (30 days), how often… “Have you had thoughts of killing yourself?”, “Have you made plans of killing yourself?” and “Have you attempted killing yourself?”. A four-point Likert response format was used with responses ranging from “never” [0], “once” [1], “twice” [2], to “more than two times” [3]. A composite score was obtained by adding the three items (0–9). A score of 1 or more represented the presence of current suicidal behavior.

Substance use (alcohol, tobacco, marijuana) was measured with single-item questions on lifetime and current substance use developed by the researchers based on a review of prior literature. Items included: “Have you ever drank alcohol in your lifetime?”, “Have you ever smoked/used tobacco products in your lifetime?”, “Have you ever smoked/consumed marijuana products (toffee, stew, drink, etc.) in your lifetime?” A “yes” [1] and “no” [0] response format was used. A composite lifetime substance use was computed by adding scores on the three items (0–3) with a score of 1 or more representing the presence of lifetime substance use. Current substance use items included: In the past 30 days, how often: ”Do you drink alcoholic beverages?”, “Do you smoke/use tobacco products?” and “Do you smoke/consume marijuana products (toffee, stew, drink, etc.)?” A four-point Likert response format was used with responses ranging from “never” [0], “twice” [2], to “more than two times” [3]. A composite score of current substance use was computed by adding scores on the three items (0–9) with a score of 1 or more representing current substance use.

Sleep problems were measured with the insomnia severity index [30]. This questionnaire has seven items, which measure sleep disturbance severity, sleep-related satisfaction and the degree of daytime functional impairment, impairment in perception, and distress and concern related to the sleeping problem. Each item is rated on a 5-point Likert scale (0–4), and results were summed to provide a total score ranging from 0 to 28. A score of 0 to 7 is classified as no clinically significant insomnia, and a score of 8 to 28 is classified as a presence of insomnia. In this study, the scale reported good reliability with Cronbach’s alpha of 0.90.

Other risks and protective factors (loneliness, physical activity, pica, body image satisfaction, food insecurity, history of childhood abuse, partner support, history of intimate partner violence, current intimate partner violence, family and friends’ support) were measured with single items developed by the researchers based on previous literature. Some examples include: “Are you satisfied with your body image/shape? “Does your partner currently abuse you (physical, verbal, sexual, neglect, etc.)?”, “Have you ever been abused (physical, verbal, sexual, neglect, etc.) as a child?”, “Do you currently receive support (emotional, physical, spiritual, etc.) from your family?” and “Do you currently receive support (emotional, physical, spiritual, etc.) from your friends?” A two-point response format (“yes”—1 and “no”—0) was used for these questions.

Demographic characteristics, such as age, marital status, stage of pregnancy, income level and employment status, were also examined.

### 2.3. Data Collection Procedure

Ethical clearance was obtained from the Ethics Review Committee of the Ghana Health Service (GHS-ERC 005/05/19) and Queen’s University Health Sciences and Affiliated Teaching Hospitals Research Ethics Board (HSREB, EPID-678-19). Gatekeeper’s letters were sent to the administrators of the two participating hospitals to obtain permission. After permissions were granted, the outpatient departments of both antenatal clinics were visited by the researchers. The aims and objectives of the study were explained to all pregnant women. They were made aware that participation in the study was voluntary and that they could withdraw from the study at any time during the data collection process without any penalty. The participants were not compensated. The participants were also assured of anonymity and confidentiality as no names or other identifying details (dates of birth or addresses) were collected. Those who voluntarily agreed to take part in the study signed an informed consent form. Interviewer-administered questionnaires (uploaded on Tablets) were used to collect the data between May and June 2019.

### 2.4. Statistical Analyses

Data entry and analyses were done using the SPSS 25. Descriptive and inferential statistics, including frequencies, percentages, chi-squared tests and logistic regression analyses, were used to answer the research questions. For the logistic regression, separate models were tested for the individual predictors of depression, anxiety and suicidal behaviors in the unadjusted models. In the adjusted models, the Enter method was used, and all the predictors were entered into the models predicting each of depression, anxiety and suicidal behaviors, respectively. All the variables (confounding) controlled for in the adjusted models were based on their significant associations with mental health problems as reported in previous literature, e.g., [4,7,8,9,19,20,21,22,23,24,25,26]. In each adjusted model, the influence of a particular predictor was examined by controlling for all other predictors. The same procedure was repeated for all three dependent variables. A *p*-value < 0.05 was considered statistically significant.

## 3. Results

### 3.1. Demographic Profile of the Participants

About 250 pregnant women were contacted between the study period and 214 of them, representing a response rate of 85.6%, took part in the study. The majority (*n* = 117, 54.7%) of participants were between the ages of 26 and 35. About 67% (*n* = 143) were married, 58.9% (*n* = 126) were self-employed, and 59.8% (*n* = 128) reported an average monthly income of less than GHC500 (USD 100). It was also observed that the majority (*n* = 134, 62.6%) of the participants were in their 3rd trimester, with 29.4% (*n* = 63) and 7.9% (*n* = 17) of the participants reporting being in their 2nd and 1st trimesters, respectively.

### 3.2. Prevalence of Depression, Anxiety and Suicidal Behaviors

Participants in the study reported prevalence rates of 50.5% (95% CI = 43.6% to 57.4%), 35.5% (95% CI = 29.1% to 41.9%) and 3.3% (95% CI = 0.9% to 5.7%) for prenatal depression anxiety and current suicidal behaviors, respectively.

### 3.3. Bivariate Analyses of Factors Associated with Depression, Anxiety and Suicidal Behaviors

Results from bivariate analysis with Chi-square (and Fisher’s Exact tests where required due to small cell sizes) (Table 1) indicate that average monthly income (χ^2^ = 10.14, *p* < 0.01), planning of pregnancy (χ^2^ = 6.47, *p* < 0.05), insomnia (χ^2^ = 12.14, *p* < 0.001), pica (χ^2^ = 7.03, *p* < 0.01), body image satisfaction (χ^2^ = 23.30, *p* < 0.001), partner support (χ^2^ = 4.73, *p* < 0.05), intimate partner violence (χ^2^ = 6.24, *p* < 0.05) and family support (χ^2^ = 3.66, *p* < 0.05) were significantly associated with depression among the participants. Marital status (χ^2^ = 7.56, *p* < 0.05), planning of pregnancy (χ^2^ = 3.96, *p* < 0.05), insomnia (χ^2^ = 14.23, *p* < 0.001), lifetime suicidal behavior (χ^2^ = 12.65, *p* < 0.001), loneliness (χ^2^ = 3.63, *p* < 0.05), body image satisfaction (χ^2^ = 7.40, *p* < 0.01), childhood abuse (χ^2^ = 4.84, *p* < 0.05), partner support (χ^2^ = 8.44, *p* < 0.01), intimate partner violence (χ^2^ = 3.97, *p* < 0.05) were significantly associated with anxiety among the participants. Only lifetime suicidal behavior (χ^2^ = 25.71, *p* < 0.001) and current intimate partner violence (χ^2^ = 5.74, *p* < 0.05) were significantly associated with current suicidal behaviors among the participants.

### 3.4. Risk and Protective Factors for Prenatal Depression, Anxiety and Suicidal Behaviors

The results from Table 2 shows that the model predicting prenatal depression with all the correlates was significant (χ^2^_(26)_ = 227.45, *p* < 0.001) and explained between 27.6% and 36.8% (Cox and Snell R^2^ = 0.276 and Nagelkerke R^2^ = 0.368) of variance in depression. In the unadjusted model, average monthly income of the participants significantly predicted prenatal depression as women with an average monthly income between GHC500 and GHC1000, and those with average monthly income above GHC1000 were 58% (OR = 0.42, 95% CI = 0.21–0.87, *p* < 0.05) and 65% (OR = 0.38, 95% CI = 0.19–0.79, *p* < 0.01) *less likely* than pregnant women with an average monthly income of less than GHC500 to report prenatal depression. In the adjusted model, only pregnant women with an average monthly income between GHC500 and GHC1000 showed significant difference in their prenatal depression (AOR = 0.37, 95% CI = 0.14–0.97, *p* < 0.05) compared to pregnant women with an average monthly income of less than GHC500. Participants’ whose pregnancies were planned were 51% *less likely* to report prenatal depression (OR = 0.49, 95% CI = 0.28–0.85, *p* < 0.05) than those women with unplanned pregnancies. However, planning of pregnancy was not significantly predictive of prenatal depression in the adjusted model. In terms of psycho-behavioral factors, the presence of insomnia was associated with 2.68 increased odds of experiencing prenatal depression (OR = 2.68, 95% CI = 1.53–4.70, *p* < 0.01) and remained significant in an adjusted model (AOR = 3.06, 95% CI = 1.54–6.10, *p* < 0.01). Pica was associated with 2.41 *increased odds* for experiencing prenatal depression (OR = 2.41, 95% CI = 1.25–4.65, *p* < 0.001) and remained significant in the adjusted model (AOR = 2.62, 95% CI = 1.15–5.95, *p* < 0.01). Pregnant women, who were satisfied with body image were 76% *less likely* to experience prenatal depression (OR = 0.24, 95% CI = 0.13–0.43, *p* < 0.001) and remained significant in the adjusted model (AOR = 0.29, 95% CI = 0.14–0.62, *p* < 0.01) compared to women not satisfied with their body. For the socioenvironmental factors, pregnant women, who reported support from their partners were 74% *less likely* than unsupported women to report prenatal depression (OR = 0.26, 95% CI = 0.07–0.95, *p* < 0.05), but this finding was not significant in the adjusted model. Pregnant women, who reported intimate partner violence (IPV) had 2.60 *increased odds* for experiencing prenatal depression (OR = 2.60, 95% CI = 1.21–5.61, *p* < 0.05) than women, who had not experience IPV, but this was not significant in the adjusted model.

For the predictors of prenatal anxiety, the model with all the correlates was significant (χ^2^_(26)_ = 53.88, *p* = 0.001) and explained between 22.3% and 30.6% (Cox and Snell R^2^ = 0.223 and Nagelkerke R^2^ = 0.306) of variance in anxiety. Results from Table 2 shows that marital status of the participants significantly predicted their experience of anxiety with women, who were cohabiting reporting 10.90 *increasing odds* for anxiety compared to single/widowed women (OR = 1.34–89.01, *p* < 0.05). However, in the adjusted model, both cohabiting (AOR = 15.74, 95% CI = 1.49–166.58, *p* < 0.05) and married women (AOR = 11.89, 95% CI = 1.13–125.65, *p* < 0.05) reported *higher odds* for experiencing prenatal anxiety compared to women, who were single/widowed. Women whose pregnancies were planned were 44% *less likely* to report prenatal anxiety compared those whose pregnancies were not planned (OR = 0.56, 95% CI = 0.32–0.99, *p* < 0.05), but this association was not significant in the adjusted model. For the psycho-behavioral factors, experiencing insomnia was associated with 3.00 *increased odds* for experiencing prenatal anxiety (OR = 3.00, 95% CI = 1.68–5.36, *p* < 0.001) in both models (AOR = 3.36, 95% CI = 1.68–6.73, *p* < 0.01). Lifetime suicidal behavior was associated with 3.25 *increased odds* for prenatal anxiety (OR = 3.25, 95% CI = 1.67–6.34, *p* < 0.01) and remained significant in the adjusted model (AOR = 2.64, 95CI = 1.14–6.11, *p* < 0.05). Women, who reported being satisfied with their body image were 55% *less likely* to report prenatal anxiety (OR = 0.45, 95% CI = 0.25–0.81, *p* < 0.01) as compared with women, who were not satisfied with their body image, but this was not significant in the adjusted model. For the socioenvironmental factors, history of childhood (OR = 2.36, 95% CI = 1.08–5.16, *p* < 0.05), IPV (OR = 2.07, 95% CI = 1.01–4.27, *p* < 0.05) were associated with *increased odds* for anxiety, but not significant in the adjusted model. Women, who reported partner support were 80% *less likely* to report prenatal anxiety (OR = 0.20, 95% CI = 0.06–0.65, *p* < 0.01) and remained significant in the adjusted model (AOR = 0.17, 95% CI = 0.04–0.75, *p* < 0.05).

For the predictors of current suicidal behaviors among the participants, the model with all the correlates was significant (χ^2^_(26)_ = 61.65, *p* < 0.001) and explained between 25% and 100% (Cox and Snell R^2^ = 0.250 and Nagelkerke R^2^ = 1.000) of variance in current suicidal behaviors. Results in Table 2 shows that only current partner abuse was significantly associated with 6.50 increased odds for suicidal behaviors (OR = 6.5, 95% CI = 1.14–37.05, *p* < 0.05) compared to women who did not report current partner abuse.

## 4. Discussion

Findings from the study show a high prevalence of prenatal depression (50.5%) and anxiety (35.5%) with a low prevalence of current suicidal behaviors (3.3%). This high prevalence of prenatal mental disorders (especially depression and anxiety) represents a significant health challenge since it can predispose both mother and child to negative health and social outcomes. The rates of depression and anxiety among pregnant women reported in this study are higher than rates reported by earlier studies in Ghana [3], Africa [7,8,9] and other parts of the world [10]. This high prevalence of comorbid prenatal anxiety and depression could be due to socioeconomic disparities within Ghana’s various regions since most of the pregnant women in the study are economically disadvantaged, as reflected in their average monthly income. For example, a study among pregnant women in the second-largest city (Kumasi) in Ghana found a lower prevalence (9.9%) of prenatal depression [3]. It is also possible that the instrument used to measure depression and anxiety is more sensitive than specific instruments developed for pregnant women, such as the Edinburgh postnatal depression scale.

On the other hand, the prevalence of current suicidal behaviors found among this sample is lower than the rates reported by earlier studies. A study among pregnant women in South Africa reported an 18% prevalence of suicidal ideations and behaviors, for instance, and this is much higher than our finding of 3.3% [11]. It is also lower than the prevalence rates (22.6% suicidal behaviors) reported in other parts of the world, such as Peru [12]. Women’s lower suicidal behavior rates in the current study may be due to social desirability bias as suicide is stigmatized [31,32] and suicidal attempts are criminal offenses in Ghana [33].

We further examined the risk and protective factors for prenatal mental health problems and showed that varied demographic, psycho-behavioral and socioenvironmental factors were associated with depression, anxiety and current suicidal behaviors. For example, lower average monthly income, insomnia, pica intake and negative body image satisfaction were significantly associated with depression. Increasing income was associated with decreased chances of experiencing depression, and this could be due to the financial burdens associated with pregnancy, especially when the women do not have adequate income to cater to their own needs and the needs of their child in the future. This could lead to a sense of apprehension and feelings of sadness associated with concerns over the financial obligations involved in childbirth and care. This result is consistent with previous findings showing the protective impact of income on depression among varied populations [20,34].

Women who reported satisfaction with their body image/shape were less likely to experience depression, and this confirms previous findings on how body image/shape affects women’s self-esteem and confidence, which may predispose them to depression [22,24]. Lack of adequate sleep could also predispose pregnant women to depression as hormonal and psychosocial changes may impact their sleep quality. Insomnia has been reported as a key predisposing factor for perinatal and postpartum depression [23,25]. Non-nutritious food consumption has been a significant risk factor for depression among pregnant women in this study, and this could be due to the consumption of these foods as a way of coping with the unpleasant symptoms and food cravings associated with pregnancy [35]. Evidence in the Ghanaian literature reported Pica to be a common phenomenon among pregnant women [35].

For prenatal anxiety, marital status, insomnia, lifetime suicidal behaviors and partner support were significant risk factors. Women who were married and those who were cohabiting reported increased odds for anxiety, for example. This finding contrasts with previous research, which showed marriage to be a significant protective factor against mental health problems [36]. The current finding could be due to the quality of the marital relationships among participating women as lack of relationship satisfaction could predispose the pregnant women to negative emotional experiences, such as anxiety. On the other hand, we found partner support to be a significant protective factor against prenatal anxiety, and this is consistent with previous literature on the role of social support during stressful situations [37,38]. Pregnant women who lack support from their partners may feel neglected, which could predispose them to negative emotional experiences. Future research into the impact of relationship quality on anxiety among pregnant women might further our understanding of the potential link.

Insomnia has been found to be a significant risk factor for anxiety. Lack of quality sleep due to unpleasant experiences and worries can lead to anxiety. This finding is consistent with previous studies, which found sleep problems as significant predictors of anxiety across patient groups [39,40]. Women with a history of any suicidal behavior (ideation, plan and attempt) were also more likely to experience prenatal anxiety. Suicidal behaviors are precipitated by complex psychosocial and physical challenges that could also predispose individuals to experience anxiety. Comorbidity of suicidal behaviors and other affective disorders, such as anxiety and depression, have been reported in the extant literature [11,41].

Current suicidal behaviors among participating pregnant women were significantly predicted by current partner abuse. In other words, women who reported currently being abused by their partners were more likely to report suicidal behaviors. Intimate partner violence has been identified as a significant risk factor for negative maternal and child health outcomes, including physical and psychological health [42]. These challenges, coupled with other social-environmental dynamics, could predispose pregnant women to engage in suicidal behaviors. The finding is consistent with previous research findings demonstrating the negative impact of partner abuse on women’s health and wellbeing and the potential specific burden of IPV on pregnant women across contexts [16,43].

## 5. Implications of the Findings

Findings from this study have implications for clinical practice, research and policy. Prenatal anxiety, depression, and suicidal behaviors were relatively common and had a high potential negative health burden. There is a need for mental health screening as part of routine maternal healthcare services at health facilities. This would ensure early identification of cases and target interventions or referrals for some women to seek more specialized mental healthcare. In addition, there is the need for relationship counseling during pregnancy to identify cases of abuse in spousal relationships for early psychosocial interventions. For research, there is the need for longitudinal studies to explore the mental health trajectories of pregnant women from perinatal to postpartum periods to plan appropriate intervention strategies targeted their specific mental health needs. For policy implications, there is the need for the training of maternal healthcare providers, especially midwives, in brief, psychosocial counseling skills and the provision of continued professional development in basic mental healthcare to address the unmet mental health needs of pregnant women.

## 6. Limitations

This study is limited by its relatively small size taking into consideration the sociodemographic, psycho-behavioral and socioenvironmental factors that we adjusted for in our analysis. The convenience sampling technique used could lead to respondent bias, and no causal inferences can be drawn from the results due to using cross-sectional data. Additionally, social desirability bias likely resulted in an underestimation of suicidal behaviors. It is also possible that medication use by pregnant women could influence the observed associations and, as such, may serve to limit the findings. However, the current results could help to inform further research in maternal mental health employing both qualitative and quantitative methods.

## 7. Conclusions

This study shows concerning prevalence rates of mental health problems, especially anxiety and depression, among pregnant women in Ghana. Negative mental health is directly associated with negative overall health and wellbeing among pregnant women and their unborn children. Significant risk and protective factors should be targeted for intervention programs to improve maternally and child health. This study focuses on the Ghanaian context but may have relevance in other LMIC healthcare environments where prevailing socioeconomic and political situations are similar.

## Figures and Tables

**Table 1:** Summary of descriptive statistics and factors associated with depression, anxiety and suicidality among pregnant women.

Variables	Sample	Depression (%)		Anxiety		Current Suicidal Behaviors	
	*N* = 214	(50.5%)	*p*	76 (35.5%)	*p*	7 (3.3%)	*p*
Sociodemographic characteristics							
*Age*			0.381		0.717		0.579 ^a^
18–25 y	65 (30.4%)	36 (55.4%)		24 (36.9%)		3 (4.6%)	
26–35 y	117 (54.7%)	54 (46.2%)		39 (33.3%)		4 (3.4%)	
36 y and above	32 (15.0%)	18 (56.3%)		13 (40.6%)		0 (0.0%)	
*Marital status*			0.060		**0.023**		0.635 ^a^
Single/widowed	14 (6.5%	5 (35.7%)		1 (7.1%)		0 (0.0%)	
Cohabiting	57 (26.6%)	36 (63.2%)		26 (45.6%)		3 (5.3%)	
Married	143 (66.8%)	67 (46.9%)		49 (34.3%)		4 (2.8%)	
*Employment*			0.293		0.791		0.266 ^a^
Unemployed	39 (18.2%)	20 (51.3%)		12 (30.8%)		2 (5.1%)	
Employed	49 (22.9%)	20 (40.8%)		18 (36.7%)		0 (0.0%)	
Self-employed	126 (58.9%)	68 (54.0%)		46 (36.5%)		5 (4.0%)	
*Average monthly income*			**0.006**		0.919		0.753 ^a^
Less than GHC500	128 (59.8%)	76 (59.4%)		45 (35.2%)		4 (3.1%)	
Between GHC500 and 1000	42 (19.6%)	16 (38.1%)		16 (38.1%)		2 (4.8%)	
Above GHC1000	44 (20.6%)	16 (36.4%)		15 (34.1%)		1 (2.3%)	
*Trimester*			0.123		0.841		0.147 ^a^
1st Trimester	17 (7.9%)	9 (52.9%)		7 (41.2%)		2 (11.8%)	
2nd Trimester	63 (29.4%)	25 (39.7%)		23 (36.5%)		1 (1.6%)	
3rd Trimester	134 (62.6%)	74 (55.2%)		46 (34.3%)		4 (3.0%)	
*Pregnancy planned*			**0.011**		**0.047**		0.576 ^a^
No	85 (39.7%)	52 (61.2%)		37 (43.5%)		3 (3.5%)	
Yes	129 (60.3%)	56 (43.4%)		39 (30.2%)		4 (3.1%)	
*Psycho-Behavioral factors*							
Insomnia	90 (42.1%)	58 (64.4%)	**<0.001**	45 (50.0%)	**<0.001**	4 (4.4%)	0.328 ^a^
Lifetime substance use	108 (50.5%)	57 (52.8%)	0.495	38 (35.2%)	0.919	5 (4.6%)	0.230 ^a^
Lifetime suicidal behavior	47 (22.0%)	29 (61.7%)	0.081	27 (57.4%)	**<0.001**	7 (14.9%)	**<0.001**
Current substance use	14 (6.5%)	7 (50.0%)	0.971	6 (42.9%)	0.553	1 (7.1%)	0.382 ^a^
Loneliness	75 (35.0%)	41 (54.7%)	0.367	33 (44.0%)	**0.040**	4 (5.3%)	0.243 ^a^
Physical activity	191 (89.3%)	94 (49.2%)	0.291	66 (34.6%)	0.398	7 (3.7%)	0.351
Pica (non-nutritious food consumption)	51 (23.8%)	34 (66.7%)	**0.008**	19 (37.3%)	0.766	1 (2.0%)	1.000 ^a^
Body image satisfaction	133 (62.1%)	50 (37.6%)	**<0.001**	38 (28.6%)	**0.007**	3 (2.3%)	0.430 ^a^
*Socioenvironmental factors*							
Food insecurity	15 (7.0%)	6 (40.0%)	0.400	4 (26.7%)	0.458	0 (0.0%)	1.000 ^a^
Childhood abuse	30 (14.0%)	14 (46.7%)	0.653	16 (53.3%)	**0.028**	2 (6.7%)	0.255 ^a^
Partner support	200 (93.5%)	97 (48.5%)	**0.030**	66 (33.0%)	**0.004**	6 (3.0%)	0.382 ^a^
Intimate partner violence	36 (16.8%)	25 (69.4%)	**0.013**	18 (50.0%)	**0.046**	2 (5.6%)	0.335 ^a^
Current partner abuse	14 (6.5%)	9 (64.3%)	0.285	8 (57.1%)	0.080	2 (14.3%)	**0.017**
Family support	184 (86.0%)	88 (47.8%)	**0.042**	63 (34.2%)	0.334	7 (3.8%)	0.277
Friends support	157 (73.4%)	76 (48.4%)	0.317	53 (33.8%)	0.373	5 (3.2%)	0.906

^a^ = Fisher’s exact test. NB: The columns for depression, anxiety and suicidal behaviors will not add up to 100% because each percentage represents the “caseness” within a specific category. ‘*p*’-values in bold are significant at the 0.05 or 0.01 level of significance. GHC500 was equivalent to USD100.

**Table 2:** Multivariate analyses of demographic, psycho-behavioral and socio-environmental predictors of depression, anxiety and suicidality.

Variables	Depression (%)		Anxiety		Current Suicidal Behaviors	
	OR (95% CI)	AOR (95%)	OR (95% CI)	AOR (95%)	OR (95% CI)	AOR (95%)
**Sociodemographic characteristics**						
Age						
18–25 y	1	1	1	1	1	1
26–35 y	0.69 (0.38–1.27)	1.02 (0.39–2.62)	0.85 (0.45–1.61)	0.81 (0.33–2.03)	0.73 (0.16–3.37)	---
36 years and above	1.04 (0.44–2.43)	1.33 (0.41–4.29)	1.17 (0.49–2.78)	1.12 (0.36–3.49)	---	---
Marital status						
Single/widowed	1	1	1	1	1	1
Cohabiting	3.09 (0.91–10.44)	4.97 (0.96–25.58)	10.90 (1.34–89.01) *	15.74 (1.49–166.58) *	-	---
Married	1.59 (0.51–4.97)	3.48 (0.68–17.96)	6.78 (0.86–53.33)	11.89 (1.13–125.65) *	-	---
Employment						
Unemployed	1	1	1	1	1	1
Employed	0.66 (0.28–1.53)	1.47 (0.36–6.05)	1.31 (0.53–3.20)	1.95 (0.49–7.70)	---	---
Self-employed	1.11 (0.54–2.29)	1.28 (0.48–3.46)	1.29 (0.60–2.80)	1.10 (0.40–3.04)	0.76 (0.14–4.10)	---
Average monthly income						
Less than GHC 500	1	1	1	1	1	1
Between GHC 500 and 1000	0.42 (0.21–0.87) *	0.37 (0.14–0.97) *	1.09 (0.53–2.23)	1.35 (0.53–3.44)	1.58 (0.28–8.92)	---
Above GHC 1000	0.38 (0.19–0.79) **	0.34 (0.10–1.17)	0.79 (0.38–1.67)	0.81 (0.24–2.74)	0.77 (0.08–7.07)	---
Trimester						
1st trimester	1	1	1	1	1	1
2nd trimester	0.59 (0.20–1.72)	0.41 (0.11–1.61)	0.82 (0.28–2.45)	0.65 (0.17–2.59)	0.12 (0.01–1.42)	---
3rd trimester	1.20 (0.40–3.01)	0.77 (0.22–2.74)	0.75 (0.27–2.09)	0.59 (0.16–2.19)	0.23 (0.04–1.37)	---
Pregnancy planned						
No	1	1	1	1	1	1
Yes	0.49 (0.28–0.85) *	0.87 (0.41–1.87)	0.56 (0.32–0.99)*	0.58 (0.27–1.21)	0.88 (0.19–4.01)	---
**Psycho-Behavioral factors**						
Insomnia	2.68 (1.53–4.70) **	3.06 (1.54–6.10) **	3.00 (1.68–5.36) ***	3.36 (1.68–6.73) **	1.88 (0.41–8.60)	---
Lifetime substance use	1.21 (0.71–2.06)	1.74 (0.83–3.63)	0.97 (0.56–1.70)	0.86 (0.41–1.77)	2.52 (0.48–13.31)	---
Lifetime suicidal behavior	1.80 (0.93–3.48)	1.56 (0.66–3.68)	3.25 (1.67–6.34) **	2.64 (1.14–6.11) *	---	---
Current substance use	0.98 (0.33–2.90)	0.39 (0.09–1.82)	1.39 (0.47–4.18)	1.08 (0.24–4.97)	2.49 (0.28–22.23)	---
Loneliness	1.30 (0.74–2.28)	0.81 (0.37–1.80)	1.75 (0.98–3.14)	1.30 (0.60–2.85)	2.55 (0.56–11.73)	---
Physical activity	0.62 (0.26–1.51)	0.58 (0.18–1.86)	0.69 (0.29–1.65)	0.68 (0.22–2.04)	---	---
Pica (non-nutritious food consumption)	2.41 (1.25–4.65) **	2.62 (1.15–5.95) *	1.10 (0.58–2.12)	0.93 (0.42–2.08)	0.52 (0.06–4.45)	---
Body image satisfaction	0.24 (0.13–0.43) ***	0.29 (0.14–0.62) **	0.45 (0.25–0.81) **	0.71 (0.34–1.46)	0.44 (0.10–2.04)	---
**Socioenvironmental factors**						
Food insecurity	0.63 (0.22–1.85)	0.29 (0.07–1.21)	0.64 (0.20–2.09)	0.48 (0.11–2.19)	---	---
Childhood abuse	0.84 (0.39–1.82)	0.50 (0.18–1.44)	2.36 (1.08–5.16) *	1.73 (0.64–4.67)	2.56 (0.47–13.82)	---
Partner support	0.26 (0.07–0.95) *	0.38 (0.08–1.78)	0.20 (0.06–0.65) **	.17 (0.04–0.75)*	0.40 (0.05–3.59)	---
Intimate partner violence	2.60 (1.21–5.61) *	2.54 (0.71–9.14)	2.07 (1.01–4.27) *	1.21 (0.39–3.75)	2.04 (0.38–10.93)	---
Current partner abuse	1.84 (0.60–5.67)	0.29 (0.05–1.76)	2.59 (0.86–7.76)	1.03 (0.20–5.27)	6.50 (1.14–37.05) *	---
Family support	0.46 (0.20–1.03)	0.83 (0.28–2.46)	0.68 (0.31–1.49)	1.12 (0.40–3.19)	---	---
Friends support	0.73 (0.40–1.35)	0.91 (0.39–2.14)	0.75 (0.40–1.41)	0.90 (0.38–2.10)	0.91 (0.17–4.80)	---

NB: All others variables were adjusted in the second models. * = *p* < 0.05, ** = *p* < 0.01, *** = *p* < 0.001, --- = insufficient numbers (frequencies) to compute statistics. OR = crude odd ratio, AOR = Adjusted odd ratio.

## Data Availability

Anonymized data are available upon a reasonable request from the corresponding author.
